# Chromosome-scale assembly and high-density genetic map of the yellow drum, *Nibea albiflora*

**DOI:** 10.1038/s41597-021-01045-z

**Published:** 2021-10-15

**Authors:** Dongdong Xu, Wanchang Zhang, Ruiyi Chen, Hongbin Song, Lu Tian, Peng Tan, Ligai Wang, Qihui Zhu, Bin Wu, Bao Lou, Jiumeng Min, Juhong Zhou

**Affiliations:** 1 Key Lab of Mariculture and Enhancement of Zhejiang Province, Zhejiang Marine Fisheries Research Institute, 316100 Zhoushan, China; 2 Key Lab of Aquatic Resources and Utilization of Jiangxi Province, School of Life Sciences, Nanchang University, 999 Xuefu Avenue, Nanchang, 330031 China; 3 Beijing Genomics Institute (BGI)-Shenzhen, Shenzhen, 518083 China; 4 Zhejiang Academy of Agricultural Sciences, Hangzhou, 310021 China

**Keywords:** Animal breeding, Next-generation sequencing, Agricultural genetics

## Abstract

The yellow drum (*Nibea albiflora*) is an economically important sciaenid fish in East Asian countries. In this study, we sequenced and assembled a near-complete gynogenetic yellow drum genome. We generated 45.63 Gb of Illumina short-reads and 80.27 Gb of PacBio long-reads and assembled them into a 628.01-Mb genome with a contig N50 of 4.42 Mb. Twenty-four chromosomes with a scaffold N50 of 26.73 Mb were obtained using the Hi-C analysis. We predicted a set of 27,069 protein-coding genes, of which 1,581 and 2,583 were expanded and contracted gene families, respectively. The most expanded genes were categorised into the protein binding, zinc-ion binding and ATP binding functional pathways. We built a high-density genetic linkage map that spanned 4,300.2 cM with 24 linkage groups and a resolution of 0.69 cM. The high-quality reference genome and annotated profiles that we produced will not only increase our understanding of the genetic architecture of economic traits in the yellow drum, but also help us explore the evolution and unique biological characteristics of sciaenid fishes.

## Abstract (summary)

**Table:** 

Measurement(s)	DNA • genome • sequence_assembly • sequence feature annotation
Technology Type(s)	DNA sequencing • sequence assembly process • sequence annotation
Sample Characteristic - Organism	Nibea albiflora

Machine-accessible metadata file describing the reported data: 10.6084/m9.figshare.16528335

## Background & Summary

Sciaenidae is one of the largest families within Perciformes; it consists of 66 genera and approximately 294 species^1^. Sciaenids are valued worldwide as an important source of dietary protein and are among the most expensive seafood species owing to their delicate flavour and high nutritional value. Sciaenids also possess the unique ability to produce deep drumming sounds using their sonic muscles and swim bladder. These sounds can be species- or sex-specific and enable individuals to communicate with one another. These unique characteristics make sciaenids attractive models in genetics and breeding studies, and in acoustic-related research that focusses on behaviour, mate choice, and evolution.

The yellow drum (*Nibea albiflora*) is a sciaenid found from the South China Sea to the coastal waters of Japan and Korea and is one of the most economically important marine fish in China and other East Asian countries (FishBase: www.fishbase.org). The market price of the yellow drum is similar to that of the large yellow croaker (*Larimichthys crocea*), which is another popular fish sold in local Chinese fish markets. In recent years, interest in yellow drum farming has increased to meet market demand, as native yellow drum populations have gradually depleted due to overfishing and ocean pollution^2^. Sea cage farming of this fish has rapidly spread throughout the coastal regions of Southeast China; the annual production of the yellow drum in China currently exceeds 60,000 tons^3^ and has the potential to become a large-scale industry, akin to the farming of the large yellow croaker, which is the most heavily farmed species among all net-cage-farmed marine fish^4^. Therefore, the production of high-quality fish seed is vital to the proper functioning of yellow drum aquaculture in China. Therefore, our laboratory and others have conducted basic studies on the yellow drum’s early reproductive biology, developed molecular tools to study this process, and carried out sex control experiments^5–7^. For instance, specific markers for sex identification had been developed^7,8^, and we generated gynogenetic fish and neo-males (XX males), which provide unique models for studies on sex determination in teleosts^4,6^. In particular, the mass production of all-female populations was achieved by crossing neo-males with normal females, which created mono-sex yellow drum cultures that are more profitable owing to the faster growth of females relative to males.

Whole-genome sequencing of a given species is an important and fundamental tool to address important issues in biological research as well as in aquaculture. To date, the genomes of four sciaenid fish species, the large yellow croaker^9,10^, miiuy croaker (*Miichthys miiuy*)^11^, spiny head croaker (*Collichthys lucidus*)^12^, and yellow drum^13^, have been sequenced and assembled *de novo* using current sequencing technologies (Online-only Table 1). Although these genome sequences have opened new avenues for the study of sciaenids, most of the draft genomes are simply assembled scaffolds based only on Illumina-sequenced short reads. Long-read sequencing technologies, i.e. the Pacific Biosciences and Nanopore long-read sequencing platforms, combined with tools that facilitate scaffold anchoring, can generate high-quality or near-complete chromosomal genome assemblies. Researchers have still not produced these types of high-quality chromosomal assemblies for several sciaenids, and this hinders the use of genome-wide association studies (GWAS) and the development of genomic breeding techniques that are often used to analyse the genetic basis of economically important traits that optimise yields.

In the present study, we constructed a high-quality chromosome-level reference genome using long-reads generated by the PacBio platform, short reads generated by the Illumina platform, and the Hi-C analysis. We also provide a high-density genetic linkage map based on genome sequencing of a full-sibling family. The genome assembly and linkage map, which includes annotated protein-coding genes, will facilitate the research of yellow drum population genetics and the functional genes associated with both economically important traits and sex determination in the yellow drum. Our study will ultimately accelerate the implementation of genetic improvement programmes in aquaculture as well as aid our understanding of the evolution of important biological characteristics, including the unique method of communication employed by the yellow drum and the species’ responses to environmental stresses.

## Methods

### Ethics statement

The Animal Ethics Committee of Zhejiang Ocean University and the Marine Fishery Institute of Zhejiang Province approved the experiments in this study under the process number: 2017C04003.

### Sampling and sequencing

**Fig. 1:**
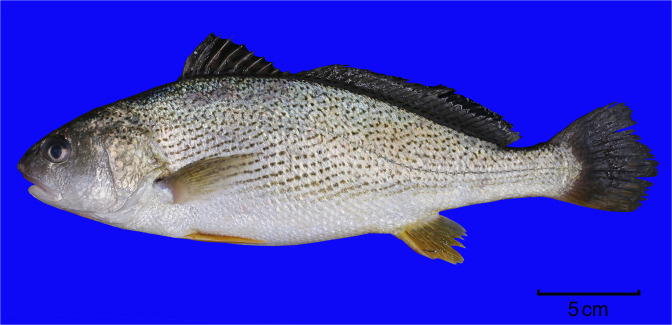
A yellow drum, *Nibea albiflora*.

DNA was isolated from the fresh muscle tissue of a gynogenetic yellow drum individual (Fig. 1) reared in the research station at the Marine Fishery Institute of Zhejiang Province (Xishan Island, Zhoushan, China). We used a standard phenol-chloroform extraction method to obtain high-molecular weight DNA. The quality of DNA was determined by gel electrophoresis to ensure that the DNA samples met library sequencing requirements. The isolated DNA was then sequenced using the PacBio Sequel II system and the Illumina HiSeq X Ten platform. According to the manufacturers’ protocols, we constructed two 20-kb libraries and sequenced them using the PacBio Sequel II platform. The Illumina HiSeq X Ten platform was used to sequence the 150-bp paired-end libraries with a 270-bp insertion. After trimming the low-quality reads and adaptor sequences from the generated data as described in^14^, we eventually obtained 45.63 Gb (70.15 ×) of Illumina short reads and 80.27 Gb (114.67 ×) of PacBio long reads for the genome assembly. The average and N50 lengths of the PacBio long subreads were 9.79 and 15.17 kb, respectively. For Hi-C library preparation, the blood sample of the same gynogenetic yellow drum individual (Fig. 1) was fixed with formaldehyde, and then the restriction enzyme *Mbo*I was added to digest the DNA as described in^15^. The 5′ overhang was then repaired using biotinylated residues. A paired-end library of approximately 300 bp insert size was constructed following standard Hi-C library preparation protocols^15^, and was sequenced on the Illumina HiSeq X Ten platform. After applying the same filtering procedures^15^, we obtained 70.42 Gb of filtered data (100.63×) and used them for genome assembly. We also collected tissue samples of the individual used for genome assembling from the following locations for PacBio full-length transcriptome sequencing: muscle, liver, heart, brain, stomach, intestine, spleen, kidney, gill, ovary, and testis. Pooled RNA samples from these tissues were used to construct two libraries, one 2 kb long and the other 5 kb long. We obtained 18.88 Gb data with an N50 length of 3.2 kb for gene annotation.

### *De novo* assembly of the yellow drum genome

We assembled a hybrid *de novo* yellow drum genome using the data generated from the Illumina and PacBio platforms and presented a description of this in Table 1. We used the PacBio long reads and Illumina short reads to correct sequencing errors in the primary data^14,16^ and estimated the genome size of yellow drums using Illumina data based on the *k*-mers spectrum. Using Jellyfish (v2.1.3)^17^, a number of 38,374,553,242 17-mers were identified. The total number of erroneous *k*-mers was 286,377,263, and the *k*-mers depth was 69. Therefore, the genome size of the yellow drum was estimated to be approximately 650.42 Mb. The proportions of repeat sequences and heterozygosity, based on the 17-mers method, were estimated to be 33.37% and 0.21%, respectively. To evaluate the PacBio SMRT sequencing reads, we used Canu (v1.5)^16^ to correct errors using the following parameters: minReadLength = 2000, minOverlapLength = 1000, -useGrid = 0, and corOvlMemory = 15. Canu was then used to perform the initial assembly of yellow drum contigs, which resulted in a 628.01-Mb genome with 1,213 contigs and an N50 of 4.42 Mb^18^.

**Table 1:** Statistical description of the yellow drum genome and annotation.

Genome assembly	Statistics
Contig N50 (Mb)	4.42
Scaffold N50 (Mb)	26.73
Scaffold N90 (Mb)	20.16
Estimated genome size (Mb)	650.42
Assembled genome size (Mb)	628.01
Number of Chromosome (N)	24
Longest chromosome length (Mb)	47.73
Average chromosome length (Mb)	25.13
Gap length (kb)	118.80

### Chromosome construction and genome quality assessment

We assembled the chromosomes using the Hi-C pipeline and obtained 147 Gb of raw data from the Hi-C libraries. We then performed quality control using HiC-Pro^19^ and mapped the 70.42 Gb of filtered data that remained to the pre-assembled yellow drum genome using Bowtie^20^ with its default parameters. We used Juicer^21^ to analyse the Hi-C datasets and the 3D *de novo* assembly (3d-dna, v. 170 123) pipeline to scaffold the 1,213 assembled contigs into 25 pseudochromosomes (24 chromosomes and 1 unknown) of lengths ranging from 8.08 to 41.21 Mb, which are consistent with the data of a previous yellow drum karyotype (2n = 48) analysis^22^. Finally, 99.84% of draft contigs were anchored into the 25 pseudochromosomes. We obtained an initial chromosomal-level yellow drum assembly with a contig N50 of 4.42 Mb and a scaffold N50 of 26.73 Mb. In another study on the yellow drum genome that was assembled using Illumina short reads, the drafted genome was estimated to be 565 Mb in length with a contig N50 of 50.3 kb and a scaffold N50 of 2.25 Mb^13^. We have aligned the Illumina short reads generated in this study to our assembled genome with default parameters and found that 98.1% of the reads were mapped to the assembly, covering 97.0% of the 24 chromosomes and 56.89% of the unplaced scaffolds, indicating high single base consistency. By taking advantage of the rapid development of sequencing and assembly techniques, we have greatly improved the assembling quality of the yellow drum genome. Compared with other studies on sciaenid genomes, our assembly yielded one of the highest assembled continuity and completeness (Online-only Table 1). We further evaluated the quality and completeness of our genome assembly using Benchmarking Universal Single-Copy Orthologs (BUSCO)^23^. Of the total 3,640 BUSCO (v4.0.6) ortholog groups (actinopterygii_odb10), 3,500 (92.3%) of which were identified in our assembled genome. Furthermore, the results placed 3,364 (92.4%) genes in the “complete single-copy” category, 56 (1.5%) in the “complete duplicated” category, 80 (2.2%) in the “fragmented” category, and 140 (3.9%) in the “missing” category.

### Repeats and gene annotations

Multiple copies of particular nucleic acids often cause an abundance of repeat sequences within a genome. We used Tandem Repeat Finder (TRF, v. 4.09)^24^, RepeatMasker (v. 3.3.0)^25^, and RepeatProteinMask (v. 3.3.0)^25^ to detect and classify different types of repetitive elements by aligning the genome sequence with the Repbase library (v. 17.01)^26^. We performed a RepeatModeler analysis of the *de novo* library and used RepeatMasker (v. 3.3.0)^25^ to categorise the transposable elements (TEs) present in the genome. We identified 127.16 Mb of repeat sequences, which accounted for 20.23% of the assembled genome. We identified 41.31 Mb of DNA TEs that dominants the most (6.52%) of the assembled genome.

**Fig. 2:**
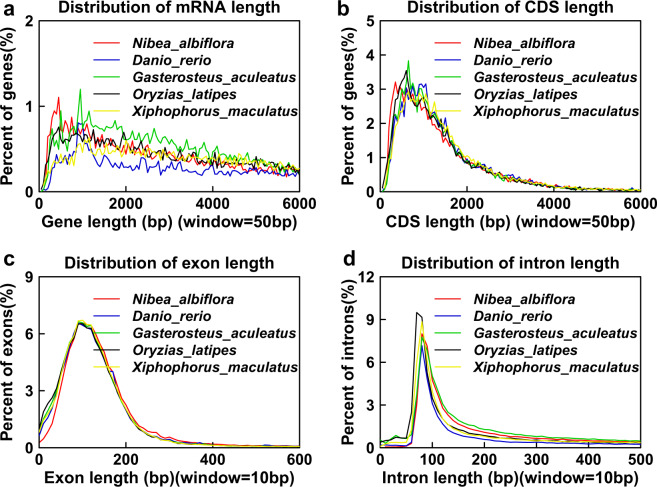
Number of orthologous genes in *N. albiflora* and 11 other species.

Structural and functional gene annotations of the assembled genome were predicted using *de novo*, homolog-based, and PacBio full-length transcriptome-based strategies. The repetitive sequences detected above were masked before the *de novo* prediction annotations were conducted. We used both Augustus (augustus-3.2.1)^27^ and Genscan^28^ to formulate *de novo* predictions, which yielded 38,857 and 38,807 protein-coding genes, respectively. The protein sequences of closely related fish species, including *Danio rerio* (26193), *Gadus morhua* (20084), *Gasterosteus aculeatus* (20772), *Oreochromis niloticus* (26763), *Oryzias latipes* (19671), *Takifugu rubripes* (18507), *Tetraodon nigroviridis* (19583), and *Xiphophorus maculatus* (20379), were downloaded from Ensembl^29^ to facilitate homolog-based gene predictions. All the protein sequences were mapped to the yellow drum genome using the Basic Local Alignment Search Tool All (BLASTALL). We predicted 23,119 genes based on the PacBio full-length transcriptomic data, which we then used in transcriptome-based annotations. After this, we integrated the results of the *de novo* predictions, homology-based annotations, and transcriptome-based annotations using GLEAN^30^, to obtain a dataset that contained 27,069 protein-coding genes. Next, we compared the number of genes, gene length, coding DNA sequence (CDS) length, exon length, and intron length distributions with those of other teleost fish species (Fig. 2).

Among the protein-coding genes, 93.89% had homologs in protein databases, including Swissprot (release-2017_09) and TrEMBL (release-2017_09), which were classified into functional categories based on the InterproScan^31^ and Kyoto Encyclopedia of Genes and Genomes (KEGG)^32^ pathway databases, as well as the Gene Ontology (GO) database^33^ (Table 2). Fisher’s exact tests were used to test for over-represented among functional categories in GO term enrichment. Multiple tests were done by using FDR (false discovery rate) correction to adjust the *P*-values.

**Table 2:** Annotation of protein-coding genes in the yellow drum.

Genome assembly		Number	Percent (%)
Total		27,069	100
Annotated	InterPro	22,383	82.69%
	GO	17,156	63.38%
	KEGG	21,892	80.87%
	Swissport	22,656	83.70%
	TrEMBL	25,344	93.63%
Unannotated		1653	6.11%

### Gene family identification and specific gene families of the yellow drum

**Fig. 3:**
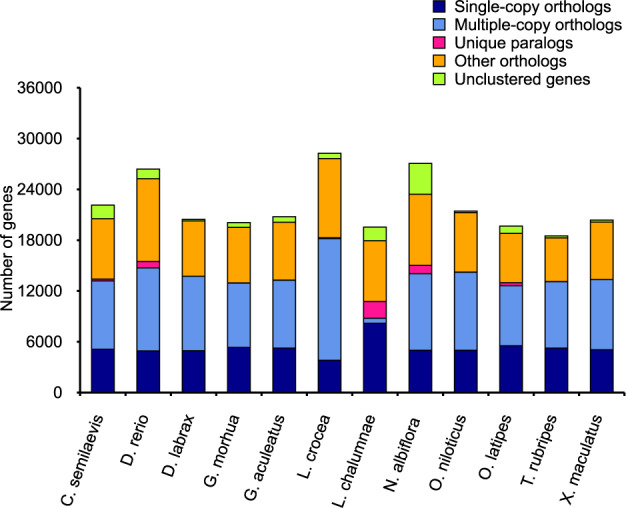
Associated statistics for the annotated gene models, including: total number of genes, CDS, and exon and intron length for the yellow drum and other related teleost species. Total gene number, CDS, and exon and intron length were compared with those of related species, including *D. rerio*, *G. aculeatus*, *O. latipes*, and *X. maculatus*.

Pairwise sequence comparisons were performed to predict orthologous genes at the genome level. In this study, we used Treefam^34^ and the BLAST approach to detect orthologous genes in the yellow drum, with a cut-off E-value of 1e-5 and a percent match cut-off of 80. Markov chain clustering was also used with the default inflation parameter in an all-to-all BLASTP analysis of entries from the other 11 teleost species, including *C. semilaevis*, *D. rerio*, *D. labrax*, *G. morhua*, *G. aculeatus*, *L. crocea*, *L. chalumnae*, *O. niloticus*, *O. latipes*, *T. rubripes*, and *X. maculatus*. We identified 27,069 genes, including single-copy and multiple-copy genes, unique paralogs, orthologs, and unclustered genes (Fig. 3). Comparisons between *N. albiflora* and *D. labrax*, *D. rerio* and *G. aculeatus* revealed 10,730 common gene families and 1,289 yellow drum-specific gene families. Additionally, the results showed that 3,648 genes that belonged to 287 gene families were specific to the yellow drum. Gene Ontology (GO) term enrichment was used to assign the majority of genes within these specific gene families to the following GO categories: protein binding, nucleic acid binding, and integral component of membrane.

### Phylogenetic analysis and divergence times

**Fig. 4:**
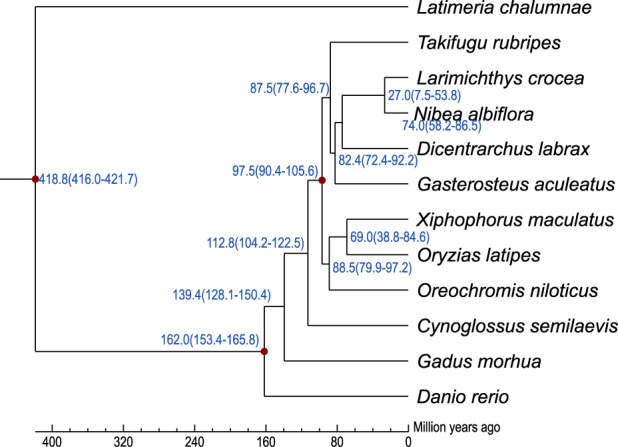
Phylogenic analysis and divergence time of the yellow drum and 11 other teleosts.

To investigate the phylogenic status of *N. albiflora*, genome-scale orthologous genes from 11 species, namely, *C. semilaevis*, *D. rerio*, *D. labrax*, *G. morhua*, *G. aculeatus*, *L. crocea*, *L. chalumnae*, *O. niloticus*, *O. latipes*, *T. rubripes*, and *X. maculatus*, were constructed to facilitate phylogenetic analyses. To eliminate redundancy caused by alternative splicing variations, we only retained the gene models at each gene locus that encoded the longest protein sequences and excluded genes that encoded protein sequences shorter than 50 amino acids since they were considered to be fragmented. We found 2,671 single-copy orthologous genes derived from entire gene families that were shared among the yellow drum and the other 11 species. We then aligned the protein sequences of multiple species in each single-copy family using MUSCLE^35^ with its default parameters and estimated the divergence time among different species using MCMCtree in PAML^36^, a correlated molecular clock, and the JC69 rate model. The phylogenetic tree showed that the yellow drum is most closely related to the yellow croaker, with an estimated divergence time of around 27 Mya (Fig. 4). In a previous study on the phylogenetic relationships among sciaenid fishes, the divergence of *Nibea* and *Larimichthys* was estimated to have occurred 16.6 Mya based on partial mtDNA and nuclear genes^37^. In the present study, we considered the divergence time among species based on whole-genome sequences to be more accurate than those based on partial genomic sequences.

### Gene family expansion and contraction analysis

Based on the inferred phylogenetic relationships and divergence times, CAFÉ^38^ (default parameters) was used to assess gene family evolution. A random birth and death model were used to study changes of gene families along each lineage of phylogenetic tree. Using conditional likelihoods as the test statistics, we calculated the corresponding *p*-values in each lineage and *p*-value of 0.05 was used to identify families that were significantly expanded and contracted. Compared with the other 11 species, the yellow drum possessed more contracted gene families (2,583) and less expanded gene families (1,581) than their common ancestor. We found that 1,581 gene families had significantly expanded in the yellow drum. Based on the functional enrichment of these expanded gene families, we identified 859 and 1,564 significant GO terms and pathways, respectively. We mainly classified these genes into eight pathways, including protein binding, zinc ion binding, and ATP binding. In addition, 667 GO terms and 945 KEGG pathways, including K04257 and K05051, were enriched among the 2,583 significantly contracted gene families. We speculate that the expanded genes contribute to the enhanced immunity that is unique to the yellow drum^9,11^.

### Synteny analysis using the large yellow croaker genome

**Fig. 5:**
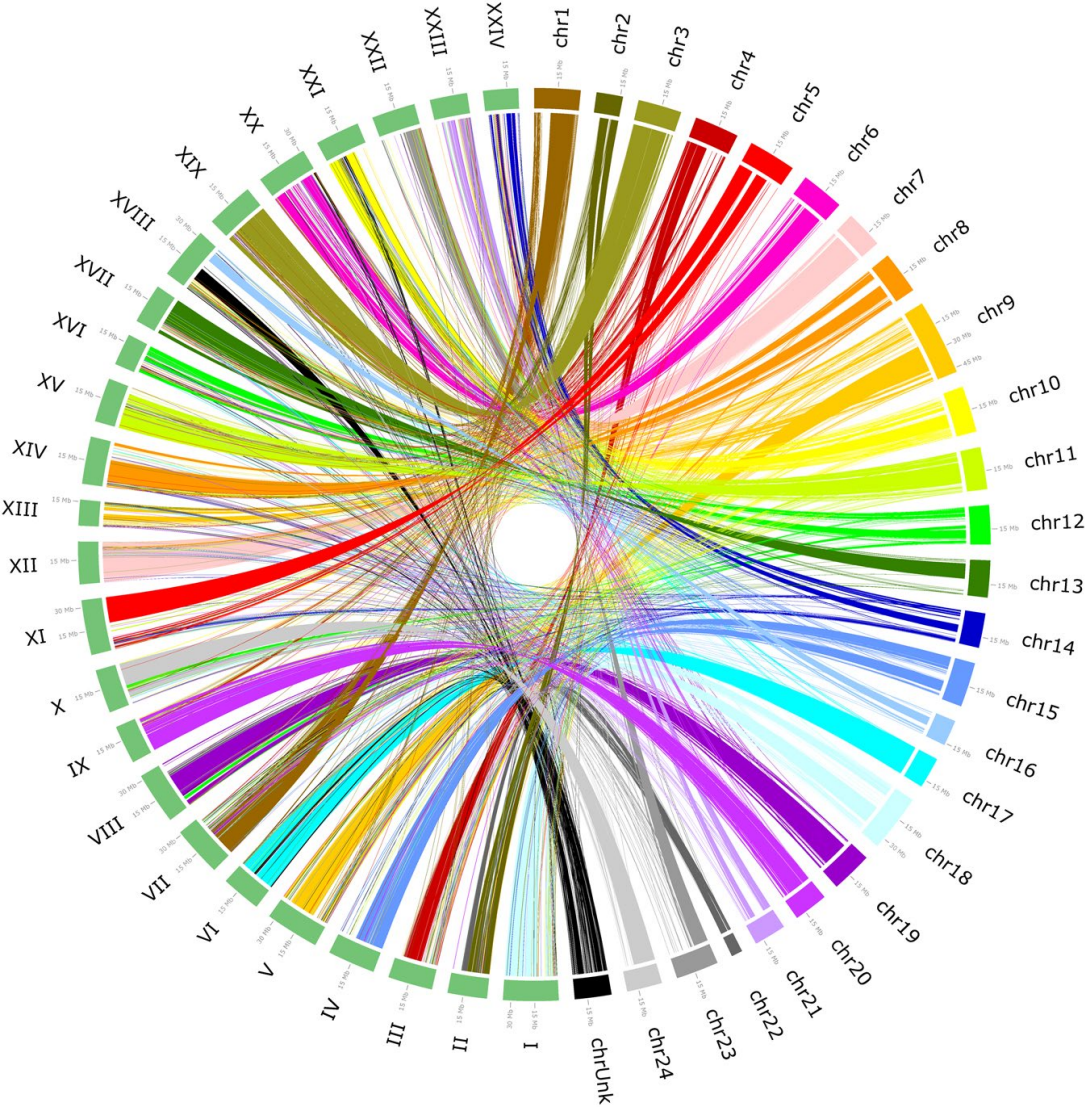
Circos plot of the synteny analysis between the yellow drum and the large yellow croaker genome.

We conducted a whole-genome synteny analysis between the yellow drum genome and the latest chromosomal-level genome of the large yellow croaker^39^ using LASTZ^40^. The whole-genome alignment between the yellow drum and the large yellow croaker genomes was visualized using Circos^41^, as shown in Fig. 5. The synteny analysis showed that 53.83% of the yellow drum genome aligned with the large yellow croaker genome (Online only Table 2). More specifically, chromosome 17 in the yellow drum and chromosome 6 in the large yellow croaker were the most highly aligned (92.63%).

### Construction of a high-density genetic linkage map

We built a full-sib F_1_ family that consisted of 200 yellow drum individuals. We sequenced the whole genomes of the parents and F_1_ offspring using the Illumina HiSeq X TEN platform. The raw reads were filtered by removing low-quality reads and adapters using Trimmomatic (v0.36)^42^. An average of 9.8 Gb of data were obtained for each individual. The clean reads were mapped to the reference assembly using “BWA-MEM” in BWA (v0.7.10-r78)^43^. Genomic alignments showed that the average sequence depths of the parents and F_1_ individuals were 45.9 × and 15.54 × , respectively.

For SNP (single nucleotide polymorphism) calling, we analysed the high-quality mapped reads of the 202 individuals using GATK (v 3.3-0)^44^. “HaplotypeCaller” was used to generate individual gVCF and then integrated by “GenotypeGVCFs”. We applied the VariantFiltration parameters as follows: “–filterExpression “QD < 2.0 || MQ < 40.0 || ReadPosRankSum <−8.0 || FS > 60.0|| HaplotypeScore >13.0 || MQRankSum <−12.5”. In total, GATK called 3.36 million raw SNPs for subsequent filtering under the following conditions: 1) GATK should only retain the bi-allele SNPs in all individuals, 2) the SNP genotype depths of parents and offspring should only be 10–30 and 5–20, respectively, 3) the genotype quality of all variants should be >10, 4) none of the SNPs in the parents should be missing and at least one of the parents should be heterozygous, and 5) the missing rate of each SNP should be <15%. Then, a total of 2,515,965 filtered SNPs was obtained, including 1,523,868 transitions and 992,097 transversions. Variant annotation classified 93,755 exonic, 1,059,586 intronic and 1,415,476 intergenic SNPs, where exonic variants consist 33,031 missense, 427 nonsense and 61,797 silent mutations. The filtered SNPs were transmitted into JoinMap4.1^45^ for the Chi-squared test (*P* < 0.05). Finally, 6,219 SNPs were used to construct a linkage map in Joinmap4.1. Twenty-four linkage groups were clustered using 6,219 SNP markers across the yellow drum genome, with the number of SNPs on each chromosome ranging from 179 to 359. Each linkage group contained an average of 260 SNP markers. The maximum likelihood method was used to sort the markers on each linkage group and estimate the genetic distances between them. The consensus map spanned 4,300.2 cM and covered nearly the whole genome with a resolution of 0.69 cM. Compared with the previous linkage map constructed by Qiu *et al*.^7^, we used more individuals with more genetic markers and produced a genetic linkage map with a higher resolution. This high-density linkage map can be used to facilitate ongoing marker-assisted selections and genomic studies on the yellow drum.

## Data Records

The PacBio, Illumina, and Hi-C sequencing data that were used for genome assembly have been deposited in the NCBI Sequence Read Archive and in GenBank under accession numbers SRR10318218^46^, SRR10799905^47^, and SRR10799906^48^. The PacBio full-length transcriptomic sequencing data were stored under accession number SRR11638044^49^. The whole genome shotgun project has been deposited at DDBJ/ENA/GenBank under the accession JABGLX000000000^50^. The chromosomal assembly, annotated profiles, original genetic map, phylogenetic tree, synteny analyses, and phenotypic records of the 202 yellow drum have been deposited in *figshare* with the publication links^51–55^.

## Technical Validation

The integrity of the extracted DNA was checked using agarose gel electrophoresis, and the main band was approximately 50 kb long, which satisfies the demands of the 20-kb insert library of the PacBio Sequel sequencing platform. The concentration of DNA was determined using a Qubit Fluorometer (Thermo Fisher Scientific, USA) and a NanoDrop ND-1000 spectrophotometer (LabTech, USA), and the absorbance was approximately 1.8 at 260/280.

For the transcriptome analysis, the total RNA was extracted using the TRIzol reagent (Invitrogen, Thermo Fisher Scientific, USA) following the manufacturer’s protocol. RNA integrity was evaluated using the Agilent 2100 Bioanalyzer (Agilent Technologies, Santa Clara, CA, USA). The samples with an RNA integrity number (RIN) of ≥7 were subjected to subsequent analyses.

## Data Availability

All commands and pipelines used in data processing were executed according to the manual and protocols of the corresponding bioinformatics software.

## Online-only Tables

**Online-only Table 1:** Summary of the assemblies and annotations of several *Sciaenid* fish genomes.

Genome feature	Larimichthys crocea^9^	Larimichthys crocea^10^	Miichthys miiuy^11^	Collichthys lucidus^12^	Nibea albiflora^13^	Nibea albiflora*
Assembled sequences (bp)	643,981,144	678,964,076	619,300,777	877,428,965	565,299,463	628,130,494
Contig N50 size (bp)	25,711	63,110	73,323	1,098,566	50,300	4,421,865
Contig N90 size (bp)	6,585	14,160	13,596	152,174		215,633
Scaffold N50 size (bp)	498,737	1,034,540	1,145,539	35,900,000	2,254,189	26,729,459
Scaffold N90 size (bp)	100,943	285,329	82,882			20,155,850
GC content (%)			40.96	42	41.8	42.61
Avg. coding DNA sequence length (bp)	1491	1,766	1,789		1,844	1,561
Benchmarking Universal Single-Copy Ortholog (%)				97.6	97.7	92.3

*The present study.

**Online-only Table 2:** Whole genome alignment results between the genome of the yellow drum (*Nibea albiflora*) and the large yellow croaker (*Larimichthys crocea*).

Chromosome of the yellow drum	Total length (bp)	Best match in the large yellow croaker chromosomes	Alignment length (bp)	Coverage (%)
1	28,993,770	VII	15,505,145	53.48%
2	16,663,570	II	9,240,440	55.45%
3	27,493,751	XIX	21,149,698	76.93%
4	27,960,023	III	11,826,427	42.30%
5	29,955,022	XI	16,528,515	55.18%
6	27,071,202	XX	14,480,688	53.49%
7	23,659,324	XII	18,757,016	79.28%
8	26,729,459	XIV	14,257,898	53.34%
9	47,731,074	V	21,189,745	44.39%
10	29,213,361	XXI	11,093,766	37.97%
11	26,531,260	XV	16,961,513	63.93%
12	24,620,309	XVI	12,696,102	51.57%
13	23,222,488	XVII	10,433,022	44.93%
14	21,758,424	XXIV	7,981,200	36.68%
15	28,077,900	IV	14,196,944	50.56%
16	16,255,354	XVIII	6,170,456	37.96%
17	19,564,411	VI	18,122,787	92.63%
18	36,185,908	I	16,040,007	44.33%
19	25,823,092	VIII	17,027,123	65.94%
20	21,377,824	IX	16,360,606	76.53%
21	20,155,850	XXIII	8,262,898	41.00%
22	6,948,392	XXII	5,105,376	73.48%
23	26,849,991		9,557,319	35.60%
24	22,182,248	X	13,346,593	60.17%
Un	23,106,487	XVIII	11,825,562	51.18%
Total	628,130,494		338,116,846	53.83%
