# Publisher Correction: Chromosome-scale assembly and high-density genetic map of the yellow drum, *Nibea albiflora*

**DOI:** 10.1038/s41597-021-01070-y

**Published:** 2021-10-19

**Authors:** Dongdong Xu, Wanchang Zhang, Ruiyi Chen, Hongbin Song, Lu Tian, Peng Tan, Ligai Wang, Qihui Zhu, Bin Wu, Bao Lou, Jiumeng Min, Juhong Zhou

**Affiliations:** 1 Key Lab of Mariculture and Enhancement of Zhejiang Province, Zhejiang Marine Fisheries Research Institute, 316100 Zhoushan, China; 2 Key Lab of Aquatic Resources and Utilization of Jiangxi Province, School of Life Sciences, Nanchang University, 999 Xuefu Avenue, Nanchang, 330031 China; 3 Beijing Genomics Institute (BGI)-Shenzhen, Shenzhen, 518083 China; 4 Zhejiang Academy of Agricultural Sciences, Hangzhou, 310021 China

**Keywords:** Animal breeding, Next-generation sequencing, Agricultural genetics

Correction to: *Scientific Data* 10.1038/s41597-021-01045-z, published online 15 October 2021

In this article the affiliation details for Dongdong Xu, Ruiyi Chen, Hongbin Song, Lu Tian, Peng Tan, Ligai Wang, Qihui Zhu were incorrectly given as ‘Zhejiang Academy of Agricultural Sciences, 310021, Hangzhou, China’ but should have been ‘Key Lab of Mariculture and Enhancement of Zhejiang Province, Zhejiang Marine Fisheries Research Institute, 316100, Zhoushan, China’. The original article has been corrected.

